# A statistical method for analyzing and comparing spatiotemporal cortical activation patterns

**DOI:** 10.1038/s41598-018-23765-w

**Published:** 2018-04-03

**Authors:** Patrick Krauss, Claus Metzner, Achim Schilling, Konstantin Tziridis, Maximilian Traxdorf, Andreas Wollbrink, Stefan Rampp, Christo Pantev, Holger Schulze

**Affiliations:** 1 Experimental Otolaryngology, University Hospital Erlangen, Friedrich-Alexander University Erlangen-Nürnberg (FAU), Erlangen, Germany; 2 Department of Physics, Center for Medical Physics and Technology, Biophysics Group, Friedrich-Alexander University Erlangen-Nürnberg (FAU), Erlangen, Germany; 3 Department of Otorhinolaryngology, Head and Neck Surgery, University Hospital Erlangen, Friedrich-Alexander University Erlangen-Nürnberg (FAU), Erlangen, Germany; 4 Institute for Biomagnetism and Biosignalanalysis, Münster University Hospital, University of Münster, Münster, Germany; 5 Department of Neurosurgery, University Hospital Erlangen, Friedrich-Alexander University Erlangen-Nürnberg (FAU), Erlangen, Germany

## Abstract

Information in the cortex is encoded in spatiotemporal patterns of neuronal activity, but the exact nature of that code still remains elusive. While onset responses to simple stimuli are associated with specific loci in cortical sensory maps, it is completely unclear how the information about a sustained stimulus is encoded that is perceived for minutes or even longer, when discharge rates have decayed back to spontaneous levels. Using a newly developed statistical approach (multidimensional cluster statistics (MCS)) that allows for a comparison of clusters of data points in n-dimensional space, we here demonstrate that the information about long-lasting stimuli is encoded in the ongoing spatiotemporal activity patterns in sensory cortex. We successfully apply MCS to multichannel local field potential recordings in different rodent models and sensory modalities, as well as to human MEG and EEG data, demonstrating its universal applicability. MCS thus indicates novel ways for the development of powerful read-out algorithms of spatiotemporal brain activity that may be implemented in innovative brain-computer interfaces (BCI).

## Introduction

Brains use sensory systems to generate internal representations of the world. These internal representations serve animal organisms as a frame of reference to guide their behavior. Nevertheless, these internal representations of the sensory surround are not a mere copy of the world, but rather models that represent certain aspects of it that are selected by sensory and cognitive filters. The available information is thereby reduced to a fraction momentarily considered as relevant, and only this part may then be perceived.

Central sensory systems realize this complex task by a combination of parallel and sequential processing of sensory information within neuronal networks, in interaction with cognitive, affective and motivational systems. It seems common sense that the internal sensory representations that result from such processing are spatiotemporal cortical activation patterns. In particular, spatiotemporal patterns of neuronal activity distributed over large numbers of neurons have been proposed (population coding) that are able to form attractor-like non-linear dynamics^1,2^. In order to form such attractor dynamics, correlated activity in only a subset of neurons within a sensory network is sufficient, whereby the mean discharge rate does not even have to be above that of spontaneous activity: just the spatiotemporal pattern of activity would be different compared to spontaneous activity without a perceptual relevance^3–5^. Although a number of studies have described such attractor-like dynamics, e.g. in the auditory cortex, based on electrocorticogram^6–9^ or extracellular spike recordings^10^, most of these studies usually analyze stimulus evoked responses within the first hundred milliseconds after stimulus onset in comparison to long-lasting spontaneous activity^10^ or during certain “marked states” that correlate with certain behavioral states^8^. As a stimulus like a tone for example may be perceived as long as it is present, the internal representation of that stimulus must be encoded somehow in the ongoing sustained activity within sensory cortex.

There are only very few studies investigating responses to long-term stimuli at all^11^, and it seems that at least some neurons might be able to respond tonically to even such long-lasting stimuli^12^. Nevertheless, as large-scale multichannel recordings were not possible by that time, these classical studies describe single unit spiking activity only and therefore could not analyze spatiotemporal activity patterns. The reason why sustained attractor dynamics still haven’t been analyzed yet may be due to the fact that proper statistics that allow for a differentiation between sustained spatiotemporal patterns belonging to the same or different internal representations have not yet been developed. Here, we therefore first report the developing of a new statistical method to analyze multichannel sustained recordings. We then demonstrate that using this approach, stimulus-specific spatiotemporal activity patterns can be detected and significantly distinguished from each other during stimulation with long-lasting stimuli. We apply the method to cortical local field potential (LFP) data recorded within different sensory modalities of different rodent species as well as to human magnetoencephalographic (MEG) and electroencephalographic (EEG) data, demonstrating the universal applicability of this new approach. Finally, we discuss possibilities for the development of new read-out algorithms of brain activity that may be used for the construction of a novel generation of brain-computer interfaces (BCI).

## Results

**Figure 1:**
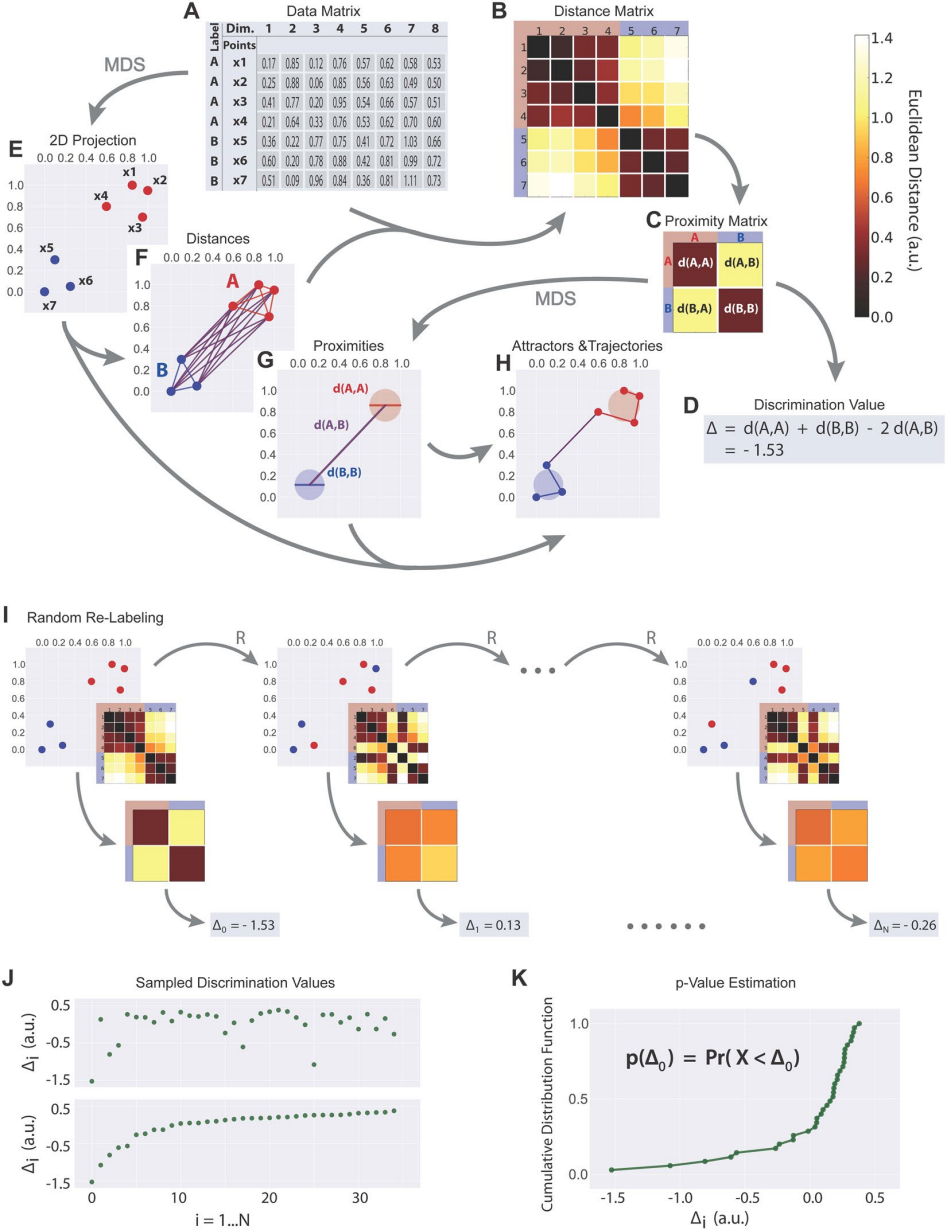
Principles of the Multidimensional Cluster Statistics (MCS): MCS allows for a statistical comparison on k clusters of data points in n-dimensional space (in this example in 8-dimensional space). For a detailed description refer to the text. Scaling of panels showing 2D-projections of data (E–H, i top panels) is in arbitrary units.

Any neurophysiological recording method provides a measure of neuronal activity that evolves over time. This measure may be a spike rate per time bin (as given in peri-stimulus time histograms) or the amplitude of the electrical potential measured via far or near field recordings, e.g. via EEG and MEG or LFP recordings, respectively. In case of multichannel recordings, such data provide a spatiotemporal pattern of neuronal activity. Consequently, we may view such data as a time series of amplitude values (x_1_ to x_i_, cf. rows in Fig. 1A) in each recording channel or dimension (Fig. 1A, columns). Furthermore, if recordings were made during a certain condition, e.g. stimulation or non-stimulation, each time bin may additionally be labeled with a marker (A, B in Fig. 1A) identifying that condition. In that sense, every spatiotemporal activity pattern that was measured during a given time bin is characterized by an n-dimensional vector, where n is given by the number of recording channels (dimensions) and the n values are given by the activity measured in each channel during that time bin. Or in other words, the spatiotemporal activity pattern may be represented by a point in n-dimensional space. Hence, as the pattern evolves over time, the point in n-dimensional space moves, forming clusters of data points that are labeled according to the recording condition. For visualization purposes, Fig. 1E shows the projection of those clusters from n-dimensional to two-dimensional space by means of multidimensional scaling (MDS^13–15^).

Until today there is no universally accepted statistical approach to evaluate whether two clusters of data points in n-dimensional space differ significantly from each other or not. Here, we present a novel statistical method to compare such clusters of data points and evaluate the method using simulated data. We then apply the method to LFP data from rodent sensory cortices of different modalities as well as human MEG and EEG data thereby demonstrating that it can be used to decode spatiotemporal cortical activity patterns.

### Analyzing clusters of data points in n-dimensional space: Theoretical derivation of the approach to compare two clusters in n-dimensional space

Our spatiotemporal multidimensional cluster statistics (MCS) method enables statistical analyzing and comparing clusters of data points in n-dimensional space. The different steps of the procedure are detailed in Fig. 1.

We start from a data matrix (Fig. 1A) containing coordinates in 8-dimensional space (columns 1 to 8) and cluster labels (in this example *A* and *B*) of all data points (x_1_ to x_7_). For visualization, these data points are projected from 8-dimensional space to 2-dimensional space by means of multidimensional scaling (MDS, Fig. 1E, cf. Methods). Next, all pairwise Euclidean distances between points are calculated (Fig. 1F) and stored in a distance matrix (Fig. 1B). Then, the mean intra- (d(A, A); d(B, B)) and inter-cluster distances (d(A, B), which is equal to d(B, A) for symmetry reasons) are derived by averaging the corresponding Euclidean distances according to the points’ cluster labels. We refer to those intra- and inter-cluster distances as proximities, which can be represented in a proximity matrix (Fig. 1C). The main diagonal entries correspond to the mean intra-cluster distances, whereas all off-diagonal entries represent mean inter-cluster distances. Furthermore, the proximities can be used to compute a discrimination value Δ (Fig. 1D) which quantifies both, the discriminability and density of the clusters of interest (cluster *A* and *B* in the example shown). We define the discrimination value of two clusters *A* and *B* as

1 $${\rm{\Delta }}({\rm{A}},{\rm{B}})={\rm{d}}({\rm{A}},{\rm{A}})+{\rm{d}}({\rm{B}},{\rm{B}})-2{\rm{d}}({\rm{A}},{\rm{B}})$$

where d(A, A), d(B, B), and d(A, B) are the mean intra- and inter-cluster distances. The last term is weighted with factor 2 to compensate for the unequal number of intra- and inter-cluster distances. The more negative this so defined discrimination value Δ is, the more distinct and/or dense the considered clusters are.

For the purpose of visualization, the off-diagonal entries of the proximity matrix (i.e. the mean inter-cluster distances) can be used to project the cluster positions (i.e. their centroids) onto a 2-dimensional plane, again by MDS (Fig. 1E, cf. Methods), and the mean intra-cluster distances (i.e. main diagonal entries of the proximity matrix) can be visualized by drawing circular areas around the projected centroids whereby the areas' diameters are chosen to be equal the corresponding mean intra-cluster distances. This leads to an abstracted visual representation of a complete spatial configuration, containing the relative positions and extent of all clusters (Fig. 1G).

In this abstracted visual representation the discrimination value of two clusters may be interpreted geometrically: it is easy to see that two clusters A and B are disjoint for Δ(A, B) < 0, whereas they are overlapping for Δ(A, B) > 0.

The temporal sequence of data points as it would result e.g. from electrophysiological recordings corresponds to a trajectory, both in state space and in projection space. Recordings under certain conditions, e.g. acoustic stimulation with a certain frequency, should result in trajectories within a restricted area in space, which may be considered as reflecting attractor dynamics. In that view, centroid positions and diameters of clusters would reflect attractor basins (Fig. 1H).

For statistical analysis of clusters we next perform N permutations of the points’ labels (i.e. random re-labeling) and re-compute the mean intra- and inter-cluster distances from which we derive the corresponding discrimination values Δ_i_ with i = 0 … N, whereby Δ_0_ corresponds to the non-permuted original labeling (Fig. 1I).

How many permutations N should be performed? With a total number of M data points and L different labels there exist L^M^ possible configurations. Hence, in most practical applications an exhaustive sampling is impossible and N has to be restricted to values typically orders of magnitude smaller than L^M^. For our analyses, where M is in a range of 200 to 400 and the number of distinct labels L is 4 in most cases, we chose N = 10^4^.

In Fig. 1J some sample discrimination values are shown, both in the order of sampling (top) and sorted by value (bottom). Using the sorted discrimination values, we compute an estimation of their cumulative probability distribution function (Fig. 1K).

The p-value to decide whether or not the analyzed clusters are significantly different from each other (i.e. significantly disjoint) corresponds to the probability of finding - by random re-labeling - a better labeling of data points in terms of smaller (i.e. more negative) discrimination values than the original Δ_0_:

2 $${\rm{p}}({{\rm{\Delta }}}_{0})={\rm{\Pr }}({\rm{X}} < {{\rm{\Delta }}}_{0})$$

This probability Pr(X < Δ_0_), and hence the p-value, can be read out directly from the cumulative probability distribution function of the discrimination values (Fig. 1K).

Note that, in principle the described MCS method would equivalently well work with the 2-dimensional projected points (Fig. 1E) instead of the high-dimensional original data points, since firstly MDS is the only dimension reduction method that preserves all mutual distances and secondly our statistical method only relies on Euclidean distances (Fig. 1B,F). Nevertheless, we apply all analyses to the original high-dimensional data and use MDS only as a tool to visualize the data points (Fig. 1E), cluster proximities (Fig. 1G), reflecting relative positions and diameters of attractor basins (Fig. 1G) and temporal trajectories (Fig. 1H).

Analogously, the described procedure can be generalized to more than two clusters (i.e. more than two distinct labels) in high-dimensional space, as described in the Supplementary Information (Supplement Fig. 1). The analytical power of our MCS approach has been tested using artificially generated data sets as detailed in Supplement Fig. 2.

### Applying the MCS method to multichannel LFP data from rodent sensory cortex

#### Analyzing sustained LFP data from gerbil auditory cortex

**Figure 2:**
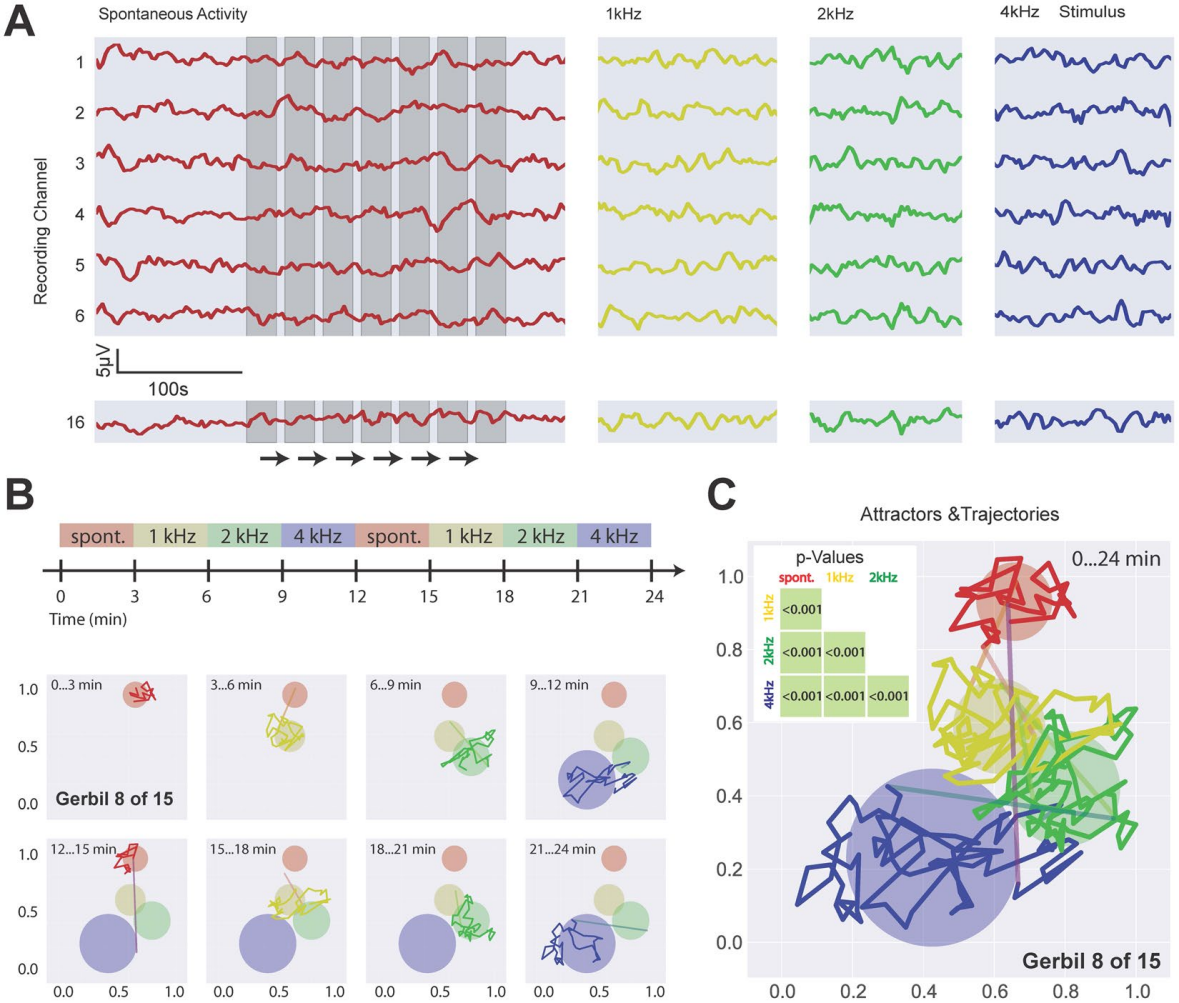
(**A**) Sample data of electrophysiological 16-channel local field potentials recorded in gerbil auditory cortex during three-minute periods of either silence (red) or the presentation of pure tones of frequencies 1, 2, or 4 kHz (yellow, green, blue). The spatiotemporal neuronal activity pattern at a given time slot (grey shaded vertical areas) is represented as a 16-dimensional state vector. In (b, top panels) the order of stimulus presentation is shown. (B, middle and bottom panels) As a function of time, the succession of state vectors form a trajectory through 16-dimensional space which is projected onto a 2-dimensional plane by multidimensional scaling. Obviously, stimulus driven neuronal attractors are not only clearly separated from the one during silence (red) but also shift systematically with stimulation frequency. Note that when stimulus conditions were repeated (b, bottom panels) activity patterns jumped back to the region where they had been during the first respective presentation (b, middle panels). Large colored dots give relative positions and diameters of attractor basins. (**C**) Overlay of all panels (b, middle and bottom). Independent from pre-stimulus activity patterns, reflected by different locations in state space, the temporal trajectories always tend to converge to, and persist, within certain regions in state space, i.e. attractors that hence are characteristic to certain stimulus conditions, large colored dots give relative positions and diameters of attractor basins associated with a certain condition. All attractors were significantly different from each other (p < 0,001, c inset). These findings were consistent in all animals tested (n = 15). Scaling of panels showing 2D-projections of data (**B**,**C**) is in arbitrary units.

We recorded electrophysiological 16-channel LFP data in gerbil auditory cortex during three-minute periods of either silence (spontaneous activity) or the presentation of tones of different frequency (1, 2, or 4 kHz). In order to apply MCS to such LFP data (Fig. 2A), we consider the spatiotemporal neuronal activity pattern at a given time slot (grey shaded vertical areas in Fig. 2A) to be reflected by a data point in 16-dimensional space, where each LFP recording channel provides amplitude values for one dimension. In other words, the LFP amplitude values within certain time windows for each recording channel can be viewed as 16-dimensional state vectors that represent the coordinates of a point in 16-dimensional space, where 16 equals the number of recording channels. Such state vectors were obtained by moving a time window through the data (20 s width, shifted in 5s steps; onset responses within the first 200 ms after stimulus onset were omitted from analysis; smoothing is applied independently on each 3 minutes stimulus time interval, cf. Supplement Fig. 3) and calculating the root-mean-square (RMS) amplitude and z-score (i.e. subtraction of mean and division by standard deviation) to remove activity common to the channels. As a function of time, the succession of data points form a trajectory through n-dimensional space (Fig. 2B,C) which reflects the dynamic changes of spatiotemporal activation patterns.

Note that during such sustained stimulation over several minutes, stimulus vs. no-stimulus conditions may not be distinguished based on neuronal discharge rate, that is, the sustained overall activity based on LFP amplitudes was not significantly different between conditions (cf. Fig. 2A, Supplement Fig. 4). MCS therefore analyzes whether the LFP activity patterns across the 16 recording channels in our array were specific to certain stimulus conditions and may be distinguished from spontaneous activity during no-stimulus conditions, even seconds or minutes after stimulus onset.

**Figure 3:**
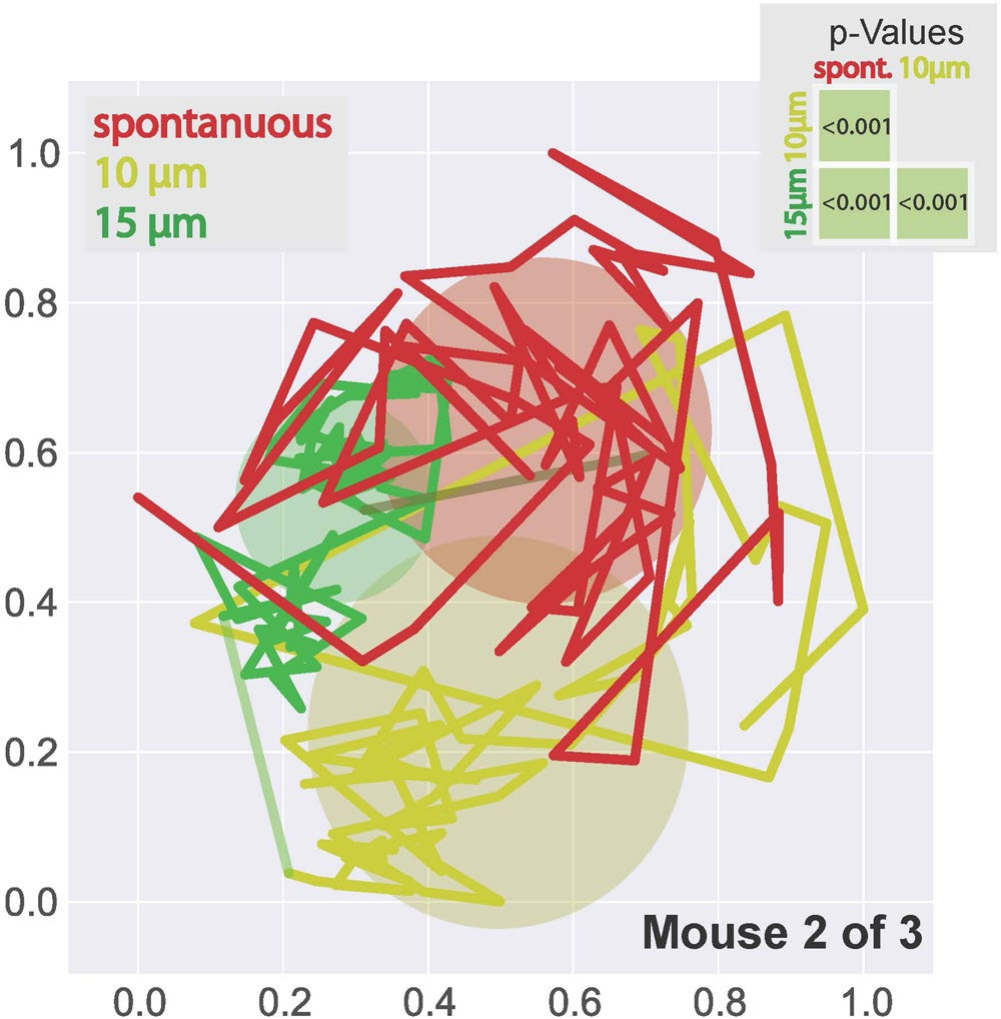
2D-projections of sample data of electrophysiological 16-channel local field potentials recorded in mouse somatosensory cortex during three-minute periods of either unstimulated (red) or stimulated conditions (yellow: vibro-tactile stimulus at 175 Hz, 10 µm oscillation amplitude; green: vibro-tactile stimulus at 175 Hz, 15 µm oscillation amplitude). All attractors were significantly different from each other (p < 0,001, inset). Scaling is in arbitrary units.

**Figure 4:**
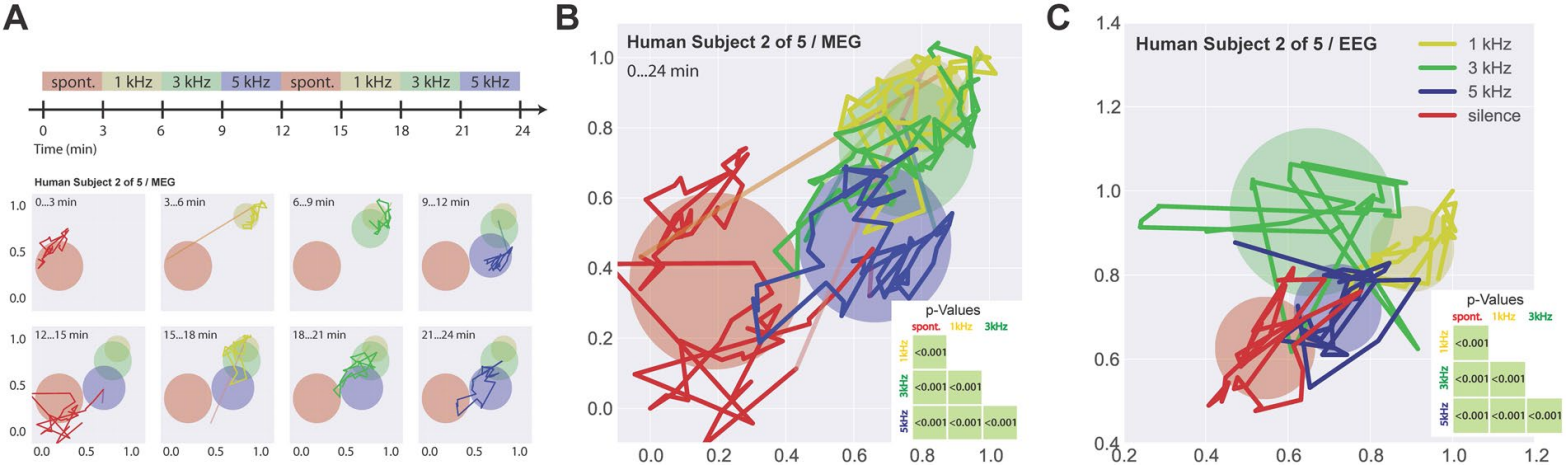
Sample data of electrophysiological 248-channel whole-head MEG recordings (**A**,**B**) and 64-channel whole head EEG recordings (**C**) in humans. Given are 2D-projections of data recorded during three-minute periods of either silence (red) or the presentation of pure tones of frequencies 1, 3, or 5 kHz (yellow, green, blue), analogously to Fig. 2. As for the animal LFP data (Figs 2 and 3), spatiotemporal cortical activity patterns in both MEG and EEG recordings showed attractor-like dynamics specific for the different stimulation conditions. All attractors were significantly different from each other (p < 0,001, insets in B and C). Scaling is in arbitrary units.

To visualize the state vectors, the corresponding data points in 16-dimensional space were projected onto a 2-dimensional target space by means of MDS which resulted in a trajectory on a 2-dimensional plane (Fig. 2B,C). Obviously, stimulus driven neuronal attractors are not only clearly separated from the one during silence (red) but also shift systematically with stimulation frequency. Note that the distance (reflecting dissimilarity of neuronal activity patterns) between the silence condition and each of the stimulus conditions is larger than between different stimulus conditions. Also note that when stimulus conditions were repeated (Fig. 2B, bottom panels; note that for this proof-of-principle study only two repetitions were performed for each stimulus condition) activity patterns jumped back to the field where they had been during the first respective presentation (cf. Supplement Fig. 5). This demonstrates that sustained LFP patterns are highly reliable and characteristic for a certain stimulus condition and by that show the attractor-like neuronal dynamics underlying these patterns. Independent from pre-stimulus activity patterns, reflected by different locations in state space and target space, the temporal trajectories always tend to converge to, and persist, within certain regions in state and target space, i.e. attractors that hence are characteristic to certain stimulus conditions (Fig. 2B, large colored dots (=attractor basins) give relative positions and diameters of clusters associated with a certain condition). It should be pointed out here that spatial relations of clusters do not reflect stimulus representations as known from topographic sensory maps (like the tonotopic map in auditory cortex) but rather reflect similarities or dissimilarities of activation patterns across the whole area of recording. As such patterns are highly individual, any pooling or averaging across individual data does not make sense, so that only individual data are shown in Figs 2–4. Using MCS it turned out that all attractors were highly significantly different from each other (p<0.001, Fig. 2C inset). Figure 2C shows a summary of this analysis (overlay of all panels of Fig. 2B). These findings were consistent in all animals tested (n = 15).

#### Analyzing sustained LFP data from mouse somatosensory cortex

In an attempt to demonstrate the robustness of the approach we tested it in another sensory modality and another animal modal, namely the mouse somatosensory cortex. Figure 3 shows the summarized outcome of such an analysis, exemplarily for one mouse (note that we measured 3 mice in total). Shown are trajectories of sustained LPF patterns and standard deviations around their centroids analogue to the diagram of Fig. 2C. Data were computed from sustained LFP recordings (onset responses omitted) during the no-stimulus condition (spontaneous activity, red), a low intensity vibro-tactile stimulus (175 Hz, 10 µm oscillation amplitude, yellow), and a high intensity stimulus (175 Hz, 15 µm oscillation amplitude, green). Again, trajectories reflecting different attractor dynamics of spatiotemporal LFP patterns can be distinguished between spontaneous activity and different stimulus conditions (all mutual p-values <0.001), demonstrating that the approach is universally applicable to different animal models and sensory modalities.

### Applying the MCS method to multichannel human MEG and EEG data

Finally, to demonstrate the applicability of MCS to even other types of measures of neuronal activity, we used the method to analyze human MEG and EEG data. Figure 4A and B show, exemplarily for one subject, the analysis of human MEG data analogously to the analysis of rodent LFP data presented in Fig. 2B and C, respectively. Data from all 248 magnetometers were used so that MCS was performed on clusters in up to 248-dimensional space and projected to 2D via MDS for visualization purposes. Again, neuronal patterns during silence (red) and stimulation with three different frequencies (1, 3, and 5 kHz, marked in yellow, green, and blue, respectively) for periods of 3 minutes each were analyzed. As for the animal LFP data, conditions during stimulation with different frequencies could be highly significantly distinguished from each other and from the silence condition (Fig. 4B, inset). Furthermore, MEG activity patterns of the different conditions were again reproducible, demonstrating the underlying, attractor-like dynamics (cf. Fig. 4A, bottom row). Similar results were obtained by analyzing 64-channel EEG data with MCS, as summarized in Fig. 4C.

## Discussion

In the present study we have demonstrated that the information about long-lasting stimuli is still encoded in spatiotemporal activity patterns in sensory cortex even after stimulus induced responses (activity changes) have subsided, that is, when overall activity is back to spontaneous levels. To do so, we have developed a new statistical method that for the first time allows for a quantitative comparison of clusters of points in n-dimensional space. By that we were able to quantify similarities and dissimilarities of cortical activation patterns across any number of recording channels. As we have further demonstrated, the method is universally applicable to any invasive or non-invasive neurophysiological recording method, from LFP to MEG, in animal models as well as in humans. However, our new statistical approach is no clustering algorithm in itself, as it was not designed to perform an unsupervised assignment^16^ of observations to different classes (clusters). Rather it compares similarities between clusters, in our case cortical activity patterns during known recording conditions that define the clusters. This is achieved by computing a discrimination value which provides an objective measure of the difference of cortical activation patterns in certain clusters, potentially reflecting the subjectively perceivable difference between stimuli.

In that context, note that a cluster in n-dimensional space does not represent a certain subarea within the brain, but rather reflects a specific group of similar activation patterns within the whole brain area that was recorded from.

Furthermore, our statistical method is not restricted to vectors of RMS amplitudes but can be applied to any kind of high-dimensional feature vectors. Thus, it can be used to evaluate classifications obtained by state-of-the-art machine learning approaches, e.g. deep neural networks and support vector machines.

Moreover, new data points can be assigned to their respective clusters with very small computational effort: the new data point x is added subsequently to each of the clusters i, and for each assignment i we compute a global discrimination value Δ(i) that quantifies how well all n clusters separate (compare Supplemental Information, Section 1). The most probable cluster is then given by minarg{Δ(i)}.

So far, a number of studies attempted to analyze spatiotemporal activity patterns but virtually none of these analyzed such patterns during sustained conditions lasting for minutes: Mazor and coworkers analyzed trajectories of populations of neurons in the locust antennal lobe and found distinct trajectories and fixed points for different stimuli^17^. Harvey and coworkers reported choice specific trajectories in parietal cortex that are not stimulus dependent, but choice dependent^18^ and population dynamics in prefrontal cortex during a choice task leading to fixed point attractors have been found by Mante^19^. Harris and colleagues analyzed patterns of spike trains across populations of neurons recorded with multichannel arrays in rat auditory cortex, but only during the first 1.5 seconds after stimulus onset^10^, so that onset responses rather than sustained activity were compared between different stimulation conditions, two types of neuronal processing that have been shown to be fundamentally different^20^. Although they report different spatiotemporal activity patterns for different stimulus conditions, they do not provide any means to statistically quantify this difference between the patterns. Furthermore, as these differences probably were strongly influenced by onset responses, they may reflect the tonotopic arrangement of neurons in auditory cortex rather than the perceptual quality of a sound that persists even if the neuronal onset responses have faded away and the overall activity is back to spontaneous levels.

Ohl and his colleagues analyzed activity patterns of electrocorticogram recordings, but restricted their analysis to certain time windows, namely peaks in dissimilarity functions between different stimulus conditions (which were largely equivalent to onset responses) in order to pre-select only those states that were maximally different before analyzing or visualizing them^8^. Again, no statement about spatiotemporal patterns associated with sustained conditions or statistical differentiation between those patterns could be made.

In our report we now overcome these limitations and for the first time present a method for statistical comparison of clusters in n-dimensional space. Our approach allowed us to distinguish between spatiotemporal neuronal activity patterns beyond those related to onset responses. These patterns were specific for a certain, sustained stimulus condition and highly reproducible.

The fact that our approach works for data from different recording techniques, representing activity from clusters of a few hundred neurons (LFP data) to that of assemblies of thousands of neurons (MEG and EEG), may be an indication of the holographic nature of the representation of information by the brain, as has first been proposed by Julesz and Pennington as well as Longuet-Higgings^21,22^.

We are therefore convinced that our approach may be used for the development of new read-out algorithms of brain activity and by that it will open new perspectives for the development of advanced brain-computer interfaces (BCI) for different applications, from game consoles to communication devices for locked-in patients.

## Methods

### Rodent data

#### Animals

Rodents (Mongolian gerbils (*Meriones unguiculatus*) and mice (*Mus musculus*, CL57 BL/6)) were housed in standard animal racks (Bio A.S. Vent Light, Zoonlab GmbH, Castrop-Rauxel, Germany) in groups of 2 (gerbils) or 4 (mice) animals per cage with free access to water and food at 20 to 24°C room temperature under 12/12 h dark/light cycle.

Gerbils were purchased from Charles River Laboratories Inc. (Sulzfeld, Germany). Mice were obtained from the Institute of Biochemistry in Erlangen.

Electrophysiological multichannel recordings were made in rodent sensory cortex, namely in auditory cortex of 15 adult male gerbils and in somatosensory cortex of 3 adult male mice.

#### Surgery

Gerbils were deeply anesthetized by a mixture of ketamine, xylacine, isotonic NaCl solution, and atropine at a mixing ratio of 9:1:8:2. Initial dose was 0.3 ml s.c., and anesthesia was maintained via s.c. infusion of the anesthetic solution, continuously applied by a syringe pump at a rate of 0.2 to 0.3 ml/h. During surgery and recording, the animal’s body temperature was kept constant at 37 °C by a remote controlled heating pad (FHC Inc., Bowdoin, ME, USA).

For recordings in gerbil auditory cortex, the skin over the skull and the musculature covering the temporal bone on the left (recording) side were partly removed. The auditory cortex was then exposed by craniotomy, leaving the dura intact. Finally, a stainless steel screw was fixed to the frontal bones with dental acrylic and served as a head anchor for stereotaxic fixation. After surgery, the still anaesthetized animals were transferred into an electrically and acoustically shielded, anechoic recording chamber.

For recordings in mouse somatosensory cortex, the animal was anesthetized by a mixture of ketavet, xylacine and isotonic NaCl solution at a mixing ratio of 3:2:15 with an initial dose of 0.2 ml s.c. The further procedure was identical to gerbils’ with the exception of the location of the trepanation lying over primary somatosensory cortex.

#### Electrophysiological LFP recordings

Acute electrophysiological multichannel recordings were made using 16-channel arrays (geometry 4 by 4, spacing 500 µm, singe electrodes with 2 MΩ impedance, 2 µm tip diameter, Clunbury Scientific, USA) inserted into the sensory cortex (auditory cortex in gerbils and somatosensory cortex in mouse). LFPs were recorded (1 kHz sampling rate, filtering: 50 Hz notch and low pass with 200 Hz upper cut-off frequency, amplification factor: 20000) during free field sound stimulation with pure tones (70 dB SPL) of different frequencies (1, 2, and 4 kHz) (gerbils) or vibro-tactile stimulation applied to the hind paw (mouse) of different intensities (LRA, C10–100 Precision Microdrives; frequency: 175 Hz, oscillation amplitudes: 10 µm and 15 µm). To obtain sustained LFP activity, recordings during stimulation were carried out continuously for 3 minutes. In addition, spontaneous activity without any stimulation was recorded for the same amount of time.

### Human data

*MEG/EEG recordings:* Simultaneous MEG (248 magnetometers, 4D Neuroimaging, San Diego, CA, USA) and EEG (64 electrodes, ASALab, ANT Neuro, Enschede, The Netherlands) were recorded (508 Hz sampling rate, filtering: 0.1–200 Hz analogue band pass, supine position, eyes open) during sound stimulation with pure tones of different frequencies (1, 3 and 5 kHz, 3 minutes each, 2 repetitions, continuous stimulation) using inset earphones (4D Neuroimaging, San Diego, CA, USA). In addition, two 3 minute runs of resting state activity were recorded. Positions of EEG electrodes and five landmarks (nasion, LPA, RPA, Cz, inion) were acquired using an integrated digitizer (Polhemus, Colchester, Vermont, Canada).

MEG data were corrected for environmental noise using a calibrated linear weighting of 23 reference sensors (manufacturers algorithm, 4D Neuroimaging, San Diego, CA, USA). Further processing was performed using Brainstorm software^23^. Data were digitally filtered (1–100 Hz, 50 Hz notch). MEG sensors and EEG electrode positions were coregistered to the ICBM 152 standard head model and atlas^24–26^ as individual MRI datasets for the participants were not available.

For estimation of noise covariance for source analysis, the first resting state recording was corrected for eye blinks and ECG artifacts based on Signal Space Projection of averaged artifact patterns, as implemented in Brainstorm. Noise covariance matrices were then estimated from the first filtered and corrected resting state data set of 3 minutes and utilized for all other recordings of the same participant. For MEG, an overlapping spheres head model, for EEG a boundary element model (BEM, OpenMEEG software^27^ was utilized. A weighted minimum-norm estimated (wMNE) was then used as inverse solution to project the measured data to the cortical surface with no orientation constraints of the sources. Time courses of activation of regions of interest (ROI) were then extracted as the mean of the activation of all source space nodes located in the respective region. All regions of the standard Desikan-Killiany atlas^28^ were selected as ROIs. The resulting data were then stored for further processing.

### Data visualization

Data are visualized using multidimensional scaling (MDS^13–15^) which can be used to project data points from a n-dimensional space onto a lower m-dimensional target space, such that all mutual Euclidean distances in n-dimensional space are preserved in target space. By that, distance in n- or m-dimensional space is a measure of dissimilarity between data points (cf. Figs 1 and 2) which in the context of this report refer to spatiotemporal patterns of neuronal activity (cf. Figs 2 to 4). Note that MDS in this report is not used as an analytical tool, but for visualization of high-dimensional data in two-dimensional data plots only. All statistics developed and presented operate in n-dimensional space and are completely independent of MDS.

### Ethics statements

The use and care of animals was approved by the state of Bavaria (Regierungspräsidium Mittelfranken, Ansbach, Germany; AZ: 54–2532.1–02/13 and 54-2532.1-42/13).

MEG/EEG data were recorded from healthy human subjects, and procedures were approved by the local ethics committees of Erlangen (registration no. 4453) and Münster (AZ: 5V Pantev: “Elektro-und Magnetoenzephalographische Korrelate neuronaler Aktivierung bei einfacher sensorischer Stimulation”). All methods were performed in accordance with the relevant guidelines and regulations. For every human participant informed consent for study participation was obtained.

### Data availability statement

All data will be made available upon request.

## Electronic supplementary material

Supplementary material

## References

[1] Dehaene S, Sergent C, Changeux JP (2003). A neuronal network model linking subjective reports and objective physiological data during conscious perception. Proc Natl Acad Sci USA.

[2] Daelli V, Treves A (2010). Neural attractor dynamics in object recognition. Exp Brain Res.

[3] Kumar A, Schrader S, Aertsen A, Rotter S (2008). The high-conductance state of cortical networks. Neural Comput.

[4] Ringach, D. L. Spontaneous and driven cortical activity: implications for computation. *Curr Opin Neurobiol* **19**, 439–444, S0959-4388(09)00078-6 (2009). 10.1016/j.conb.2009.07.005 PMC3319344 19647992

[5] Tomov P, Pena RF, Zaks MA, Roque AC (2014). Sustained oscillations, irregular firing, and chaotic dynamics in hierarchical modular networks with mixtures of electrophysiological cell types. Front Comput Neurosci.

[6] Ohl FW, Deliano M, Scheich H, Freeman WJ (2003). Early and late patterns of stimulus-related activity in auditory cortex of trained animals. Biol Cybern.

[7] Ohl FW, Deliano M, Scheich H, Freeman WJ (2003). Analysis of evoked and emergent patterns of stimulus-related auditory cortical activity. Rev Neurosci.

[8] Ohl FW, Scheich H, Freeman WJ (2001). Change in pattern of ongoing cortical activity with auditory category learning. Nature.

[9] Deliano M, Scheich H, Ohl FW (2009). Auditory cortical activity after intracortical microstimulation and its role for sensory processing and learning. J Neurosci.

[10] Harris KD (2011). How do neurons work together? Lessons from auditory cortex. Hear Res.

[11] Goldberg JM, Adrian HO, Smith FD (1964). Response of Neurons of the Superior Olivary Complex of the Cat to Acoustic Stimuli of Long Duration. J Neurophysiol.

[12] Javel E (1996). Long-term adaptation in cat auditory-nerve fiber responses. J Acoust Soc Am.

[13] Kruskal JB (1964). Multidimensional scaling by optimizing goodness of fit to a nonmetric hypothesis. Psychometrika.

[14] Kruskal JB (1964). Nonmetric multidimensional scaling: a numerical method. Psychometrika.

[15] Torgerson, W. S. Theory and methods of scaling (1958).

[16] Jain AK, Murty MN, Flynn PJ (1999). Data clustering: a review. ACM computing surveys (CSUR).

[17] Mazor O, Laurent G (2005). Transient dynamics versus fixed points in odor representations by locust antennal lobe projection neurons. Neuron.

[18] Harvey CD, Coen P, Tank DW (2012). Choice-specific sequences in parietal cortex during a virtual-navigation decision task. Nature.

[19] Mante V, Sussillo D, Shenoy KV, Newsome WT (2013). Context dependent computation by recurrent dynamics in prefrontal cortex. Nature.

[20] Maier A, Aura CJ, Leopold DA (2011). Infragranular sources of sustained local field potential responses in macaque primary visual cortex. Journal of Neuroscience.

[21] Julesz B, KS P (1965). Equidistributed information mapping-an analogy to holograms and memory. Journal of the Optical Society of America..

[22] Longuet-Higgins HC (1968). Holographic model of temporal recall. Nature.

[23] Tadel F, Baillet S, Mosher JC, Pantazis D, Leahy RM (2011). Brainstorm: a user-friendly application for MEG/EEG analysis. Computational intelligence and neuroscience.

[24] Collins, D., Zijdenbos, A., Baaré, W. & Evans, A. in *Information processing in medical imaging*. 210–223 (Springer).

[25] Fonov VS, Evans AC, McKinstry RC, Almli C, Collins D (2009). Unbiased nonlinear average age-appropriate brain templates from birth to adulthood. NeuroImage.

[26] Fonov V (2011). Unbiased average age-appropriate atlases for pediatric studies. Neuroimage.

[27] Gramfort A, Papadopoulo T, Olivi E, Clerc M (2010). OpenMEEG: opensource software for quasistatic bioelectromagnetics. Biomedical engineering online.

[28] Desikan RS (2006). An automated labeling system for subdividing the human cerebral cortex on MRI scans into gyral based regions of interest. Neuroimage.

