# Supplementary material for: A statistical method for analyzing and comparing spatiotemporal cortical activation patterns
Source: Sci Rep. 2018 Apr 3;8:5433. doi: 10.1038/s41598-018-23765-w (PMC5882928; doi:10.1038/s41598-018-23765-w)
Supplement: Supplementary file 1 — Supplementary material [file 41598_2018_23765_MOESM1_ESM.pdf]

A statistical method for analyzing and comparing spatiotemporal cortical activation patterns

Supplementary material

Patrick Krauss[1], Claus Metzner[2], Achim Schilling[1], Konstantin Tziridis[1], Maximilian Traxdorf [3], Andreas Wollbrink[4], Stefan Rampp[5], Christo Pantev[4] and Holger Schulze*[1]

[1] Experimental Otolaryngology, University Hospital Erlangen, Friedrich-Alexander University Erlangen-Nürnberg (FAU), Germany

[2] Department of Physics, Center for Medical Physics and Technology, Biophysics Group, Friedrich-Alexander University Erlangen-Nürnberg (FAU), Germany

[3] Department of Otorhinolaryngology, Head and Neck Surgery, University Hospital Erlangen, Friedrich-Alexander University Erlangen-Nürnberg (FAU), Germany

[4] Institute for Biomagnetism and Biosignalanalysis, Münster University Hospital, University of Münster, Germany

[5] Department of Neurosurgery, University Hospital Erlangen, Friedrich-Alexander University Erlangen-Nürnberg (FAU), Germany

* Corresponding author

1. Comparing k clusters in n-dimensional space

The method as described in the main text for the statistical comparison of two clusters of data points in n-dimensional space can in principal be generalized to more than two clusters (i.e. more than two distinct labels) in high-dimensional space. How this can be achieved is demonstrated in Supplement Figure 1 for the case of nine data points (x1 to x9) in 8-dimensional space (shown are only 2-dimensional projections) and three clusters or labels respectively: A, B, and C. The data matrix containing points' 8-dimensional coordinates and corresponding labels are not shown (cf. Fig. 1a).

Supplement Figure 1a shows the 2D-projection from 8-dimensional space via multidimensional scaling. For further analysis, all pairwise Euclidean distances between data points have been computed (Supplement Figure 1b) and mean intra- and inter-cluster distances (i.e. proximities) are derived by averaging corresponding pairwise points' distances (Supplement Figure 1c, d). From these mean distances again discrimination values Δ can be calculated for all pairwise cluster combinations as described above. In addition, an extended discrimination value may be derived, taking into account the combination of all three clusters (Supplement Figure 1e):

$$\Delta = d(A,A) + d(B,B) + d(C,C) - (d(A,B) + d(A,C) + d(B,C)) \qquad \text{(S1)}$$

where d(A,A), d(B,B), d(C,C), and d(A,B), d(A,C), d(B,C) are the mean intra- and inter-cluster distances.

Note that in contrast to the discrimination value for two clusters, the corresponding formula for three clusters contains no weighting factor for any term. This is because in the special case of three clusters the number of intra-cluster distances is equal to the number of inter-cluster distances.

In the general case of L clusters or labels (C_1, C_2, …, C_L) we define the discrimination value as:

$$\Delta(C_1, C_2, \dots, C_L) = \sum_{i=1}^{L} d(C_i, C_i) - \frac{2}{L-1} \sum_{i=1}^{L-1} \sum_{j=i+1}^{L} d(C_i, C_j) \qquad \text{(S2)}$$

with $d(C_i,C_i)$ the mean intra-cluster distances and $d(C_i,C_j)$ the mean inter-cluster distances. The factor 2/(L-1) compensates for different numbers of mean intra- and inter-cluster distances.

All further steps (random re-labelling of data points, evaluating the cumulative distribution function, p-value estimation) are analogously to the case of two clusters, based on the discrimination values as described in detail in Figure 1 for two clusters.

Supplement Figure 1

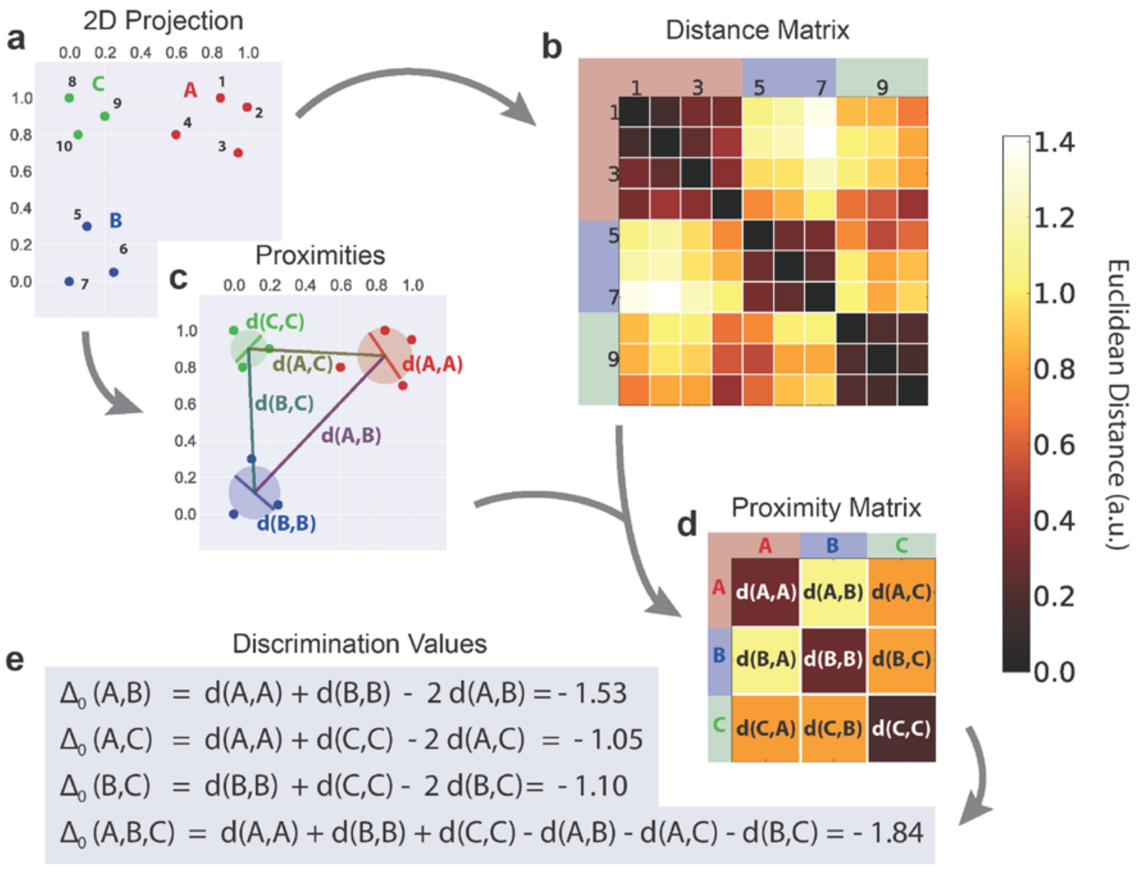

2. Evaluation of the MCS method with artificial, simulated data

In order to evaluate the analytical power of our MCS approach, we tested the method using artificially generated data sets. Supplement Figure 2 shows six examples of such simulated data sets (in 2D for simplicity) with two clusters each but different spatial patterns and degrees of overlap. In Supplement Figure 2a to c, data were generated by drawing samples from 2-dimensional Gaussian distributions with identical variances but different means, whereby distance between means decreases from a to c. Only if means of data distributions were almost identical (Supplement Figure 2c) MCS could not significantly distinguish between the two clusters anymore. In Supplement Figure 2d to f spatially more complex arrangements were tested: MCS was able to significantly separate distributions with identical means but different variances (Supplement Figure 2d), complete overlap of a Gaussian with a bimodal distribution (combined from the sum of two different Gaussians with different means but identical variances; Supplement Figure 2e) and a Gaussian distribution asymmetrically surrounded by a second cluster (Supplement Figure 2f): This c-shaped cluster (blue) in Supplement Figure 2f is a combination of 13 Gaussians, whereby their means are arranged along a c-shaped trajectory with equidistant spacing.

Supplement Figure 2

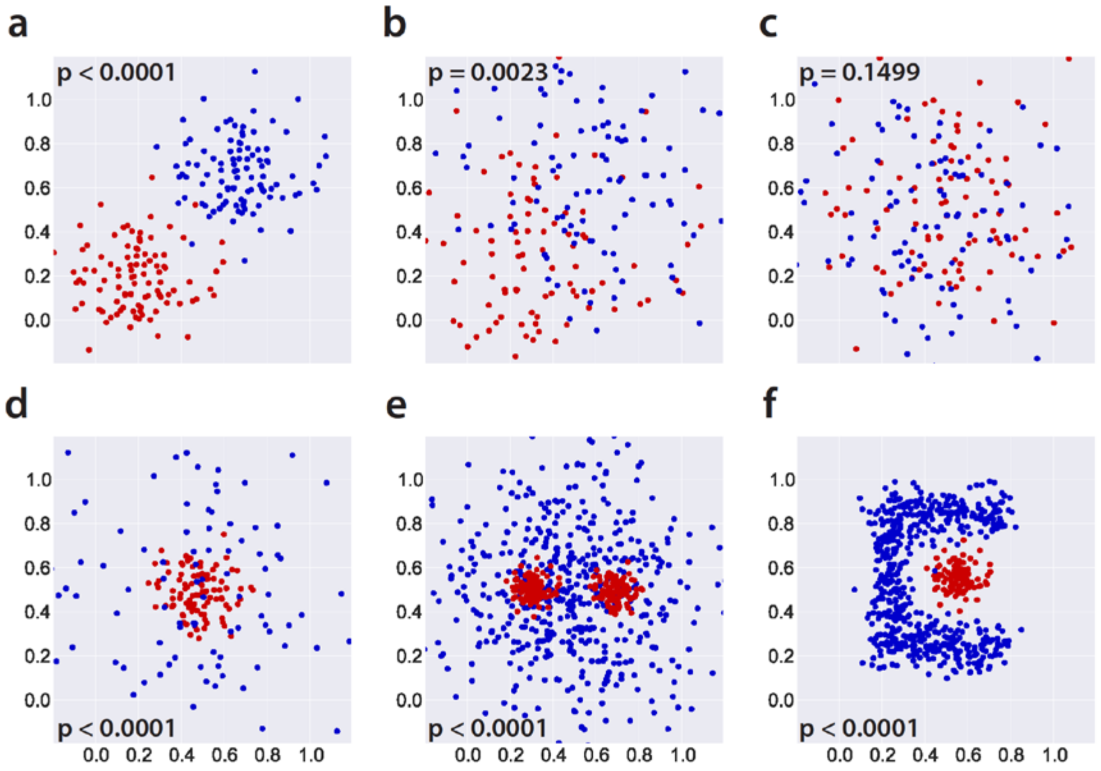

3. Effect on smoothing of LFP data

In order to demonstrate that smoothing over 20 seconds, which induces artificial correlations between data points, does not bias our results we applied our MCS method to an exemplary data set that has been smoothed only over 5 seconds intervals, thus data points are disjoint and not overlapping.

As can be seen in Supplement Figure 3 this leads to more noise projections and clusters are more overlapping. However, the clusters can still be clearly separated by eye and by our statistics (all pairwise p-values between different clusters are <0.001).

Supplement Figure 3

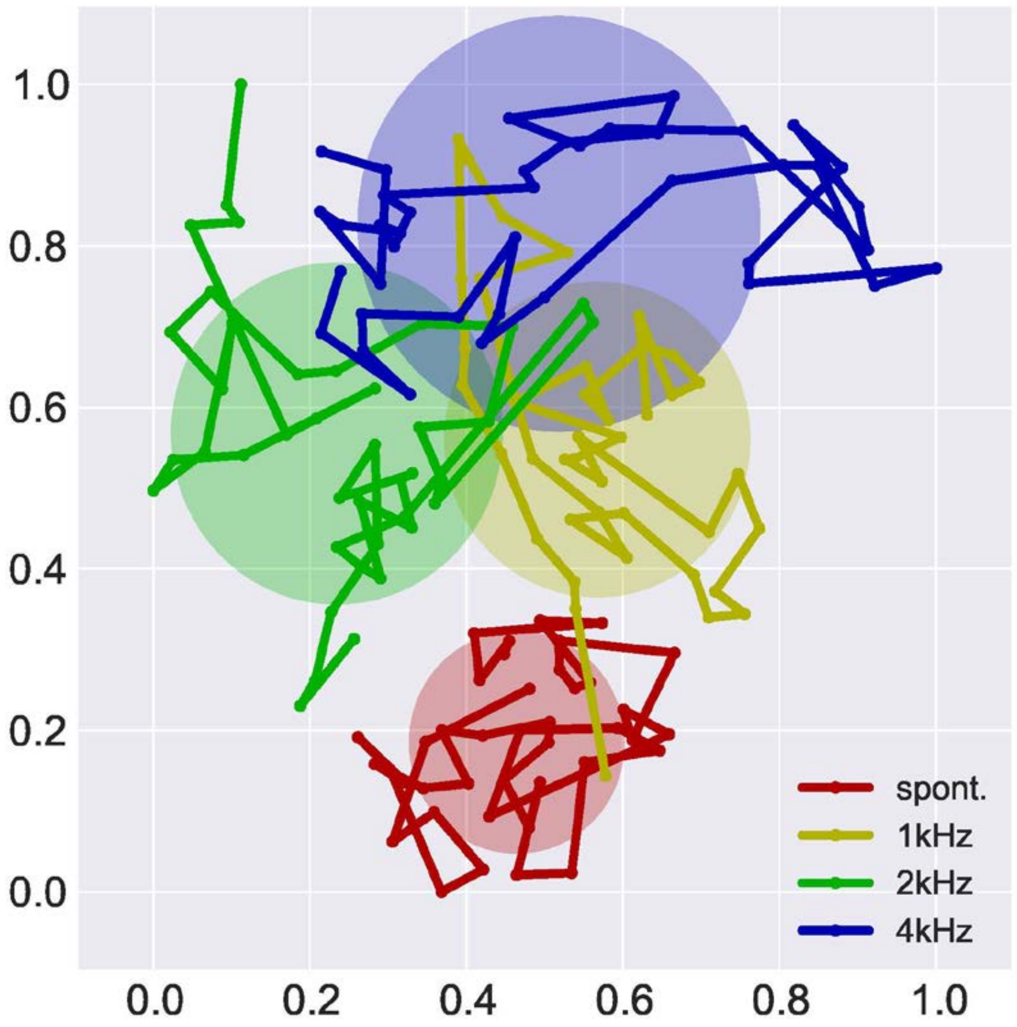

4. Classic statistical analysis of RMS data

In line with common literature[S1,S2,S3] the evaluation of the normalized (z-score) RMS values of single or multiple channels over many seconds of stimulation by classical statistical methods do not carve out any significant differences during presentation of different stimuli. This was tested exemplarily for LFP recordings with 8 electrodes located in the auditory cortex of a Mongolian gerbil. By non-parametric ANOVA analyses the LFP RMS amplitude z-scores given in Supplementary Figure 4 do neither show any significant differences over the 8-dimensional vector for each of the four stimulus conditions (Friedman-ANOVAs; p-values between p=0.78 and p=0.96) nor any significant differences between the four conditions for each individual electrode (Kruskal-Wallis-ANOVAs; p values between p=0.93 and p=0.99). Note that one could interpret the differences of the median values of the 8 electrodes over the four stimulus conditions as patterns "by eye". But these differences cannot become significant due to the huge variance of the data.

Analysis of LFP RMS amplitude z-scores by non-parametric ANOVAs. Median values (lower, upper quartile) are compared within the four different stimulus conditions (colors) by Friedman-ANOVAs and between the conditions for the 8 electrodes by Kruskal-Wallis-ANOVAs.

Supplement Figure 4

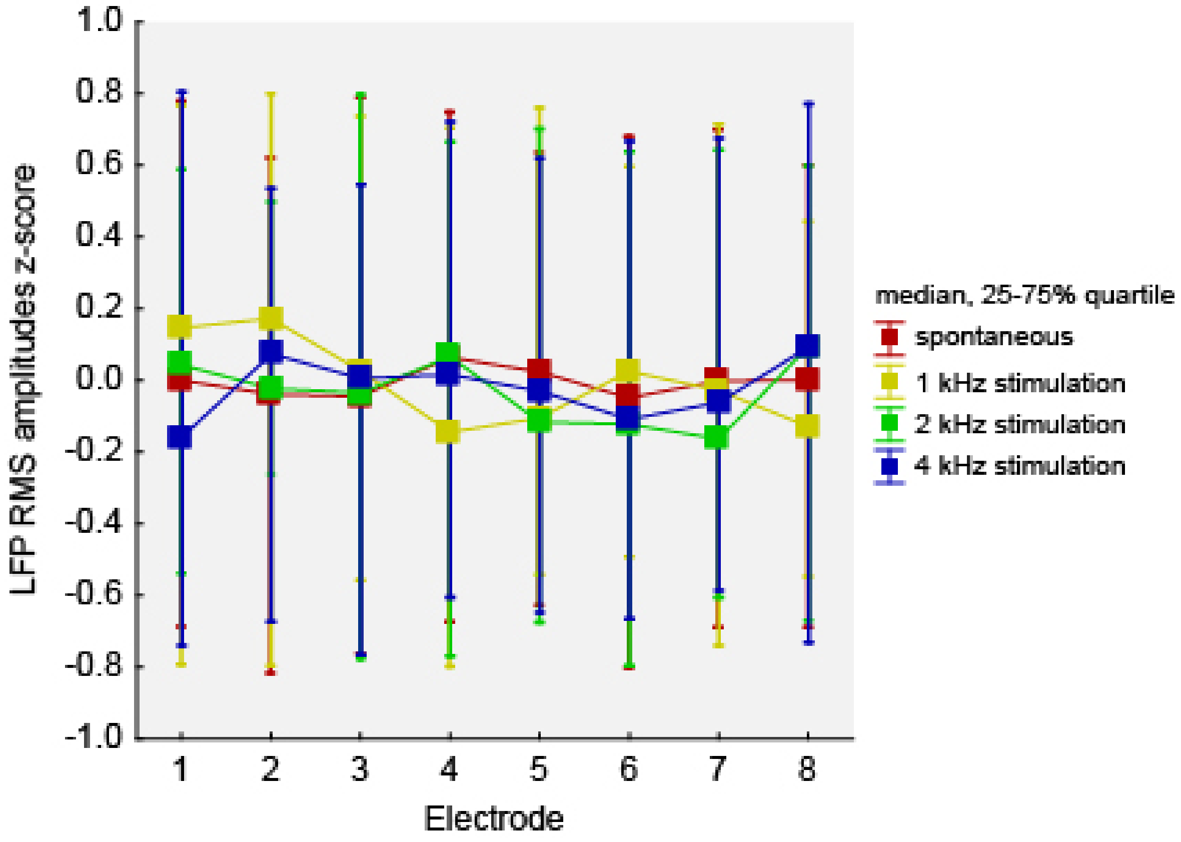

5. *Repetitive Measurements*

In order to demonstrate that our approach considers clusters from repetitions as the same cluster, we further analyzed the exemplary data shown in Figure 2. Therefore, every 3 minutes interval has been treated as a different cluster and labeled accordingly, yielding 8 clusters (4 stimulus conditions with 2 repetitions each). Evaluation of the discrimination values between each pair of clusters is summarized in Supplement Figure 5-I. Note that relatively large discrimination values indicate similarity between clusters, whereas relatively small discrimination values indicate that the two considered clusters are different.

As can be seen, the largest discrimination values are on the main diagonal of the matrix (Supplement Figure 5-I A, D). These values result from comparing each cluster with itself. Thus, this is in line with the expectation. In contrast, the off-diagonal entries of sub-matrices A and D in Supplement Figure 5-I contain relatively small discrimination values, indicating the difference of clusters of data points recorded under different stimulus conditions.

The sub-matrices B and C in Supplement Figure 5-I summarize the pairwise discrimination values from all stimulus conditions of the first trial with all stimulus conditions of the second trial. Remarkably, B and C look very similar to the aforementioned A and D. Again the largest values are located at the diagonal, whereas the off-diagonal values are small. Thus, different stimulus conditions from different trials are considered as different. But even more important, clusters from different trials recorded under the same stimulus conditions are “recognized” as very similar (though not identical) and hence belonging to the same cluster.

Supplement Figure 5-II shows the same discrimination values as 5-I, whereby those values larger than an empirically determined threshold are marked red, and values smaller than this threshold are marked blue. Only the clusters belonging to 1 KHz trial 2 and 2 KHz trial 1 are falsely classified as the same cluster.

This clearly demonstrates that our method is both, able to discriminate clusters from different stimulus conditions and correctly “recognizing” clusters from same conditions but different repetitions. Hence, our new method may be extended to a machine learning approach for automated classification of clusters.

Supplement Figure 5

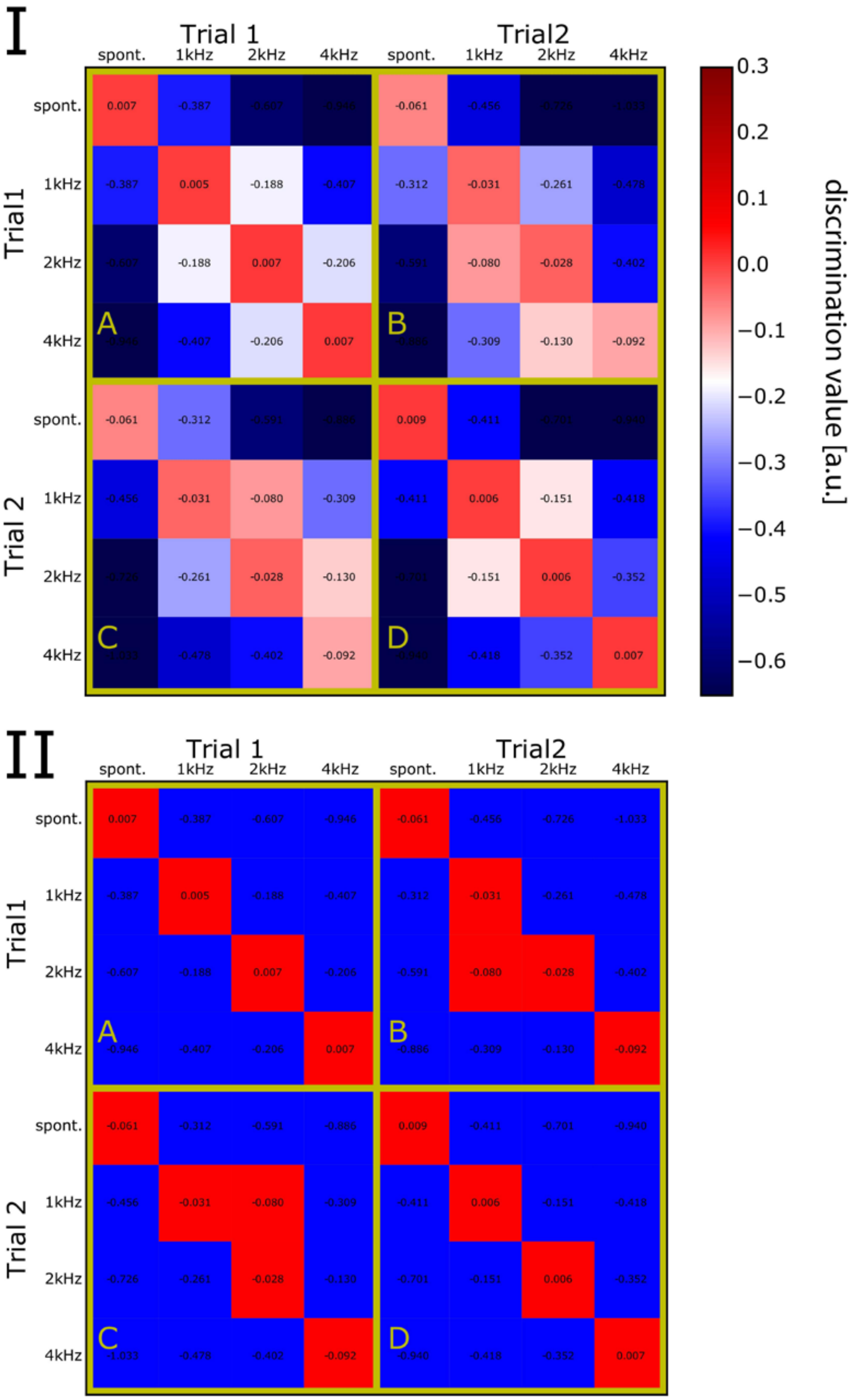

References

S1 Kumar, A., Schrader, S., Aertsen, A. & Rotter, S. The high-conductance state of cortical networks. *Neural Comput* **20**, 1-43, doi:10.1162/neco.2008.20.1.1 (2008).

S2 Ringach, D. L. Spontaneous and driven cortical activity: implications for computation. *Curr Opin Neurobiol* **19**, 439-444, doi:S0959-4388(09)00078-6 (2009).

S3 Tomov, P., Pena, R. F., Zaks, M. A. & Roque, A. C. Sustained oscillations, irregular firing, and chaotic dynamics in hierarchical modular networks with mixtures of electrophysiological cell types. *Front Comput Neurosci* **8**, 103, doi:10.3389/fncom.2014.00103 (2014).
